# Two decades of capacity building to support global malaria control and elimination: retrospective and prospective international trainings in Jiangsu Institute of Parasitic Diseases, China, 2002–2021

**DOI:** 10.1186/s12936-023-04526-1

**Published:** 2023-03-10

**Authors:** Cheng Liang, Xuedan Ke, Yuanyuan Cao, Weiming Wang, Mengmeng Yang, Jie Wang, Cecilia T. Hugo, Leonard Ortega, Glenda Gonzales, Guoding Zhu, Jun Cao

**Affiliations:** 1 Key Laboratory of National Health Commission (NHC) On Parasitic Disease Control and Prevention, Jiangsu Provincial Key Laboratory On Parasite and Vector Control Technology, Jiangsu Institute of Parasitic Diseases, Wuxi, 214064 Jiangsu China; 2 WHO Collaborating Centre for Research and Training On Malaria Elimination, Wuxi, 201064 Jiangsu China; 3 ACTMalaria Foundation, Inc. Manila, 1000 Manila, Philippines; 4 World Health Organization, Geneva, Switzerland; 5 Center for Global Health, School of Public Health, Nanjing Medical University, Nanjing, 211166 Jiangsu China; 6 World Health Organization Western Pacific Region, 1000 Manila, Philippines

**Keywords:** Malaria elimination, Capacity building, Jiangsu Institute of parasitic diseases (JIPD), Training, WHO collaborating centre

## Abstract

**Background:**

Malaria is still one of the major infectious diseases affecting human health, and the World Health Organization (WHO) has attached special importance to malaria-related technical training for its global elimination efforts. The Jiangsu Institute of Parasitic Diseases (JIPD), designated as a WHO Collaborating Centre for Research and Training on Malaria Elimination, has conducted numerous international malaria training programmes during the last 2 decades.

**Methods:**

A retrospective analysis of international training programmes organized and facilitated by JIPD in China since 2002 was conducted. A web-based questionnaire was designed to gather respondents’ basic information, evaluation of course topics, methodology, trainers, and facilitators, course impact, and suggestions for future trainings. Individuals who participated in the training courses from 2017 to 2019 were invited to participate in this assessment.

**Results:**

Since 2002, JIPD has conducted 62 malaria-related international trainings attended by 1935 participants from 85 countries, covering 73% of malaria endemic countries. Of 752 participants enrolled, 170 responded to the online survey. A majority of respondents (160/170, 94.12%) gave a high evaluation of the training, with an average score of 4.52 (5 maximum score). Also, survey respondents gave a 4.28 score on “knowledge and skills gained in the training useful for the national malaria programme”, 4.52 on “topics appropriate to their professional needs”, and 4.52 on “knowledge and skills gained in the training useful to their career”. Surveillance and response was the most important topic discussed and field visit was the most effective method of training. For future training programmes, with increasing length of training, more field visits and demonstration, improving language barrier, and sharing experience were what the respondents requested most.

**Conclusion:**

JIPD, as a professional institute for malaria control, has conducted a great quantity of training in the past 20 years, providing training opportunities to both malaria and non-malaria endemic countries globally. For future training, survey respondents’ suggestions will be considered to provide a more effective capacity building activity to better contribute to global malaria elimination.

## Background

Malaria, one of the major infectious diseases, caused approximately 241 million infections and 627,000 deaths in 85 malaria endemic countries worldwide in 2020 [1]. Countries with a relatively high burden of malaria are mainly in sub-Saharan Africa and Southeast Asia. Over the past decades, there has been a call for revitalized efforts to tackle malaria globally, and significant progress towards malaria elimination has been achieved. Even though multiple countries have launched national malaria elimination programmes and made malaria elimination a priority, challenges still remain, including: shortage of funding, insufficient technical personnel, inadequate international assistance [2].

The World Health Organization (WHO) suggests that offering relevant and timely training for malaria programme officers and health workers is of great importance and needs to be continued in all countries as long as the disease persists, regardless of the status of malaria elimination [3]. The WHO has always highlighted the importance of malaria-related technical training. One report on a teaching workshop conducted at the Malaria Eradication Training Centre in Nigeria in 1967 indicated that various forms of training could provide formal instruction for teaching staff in methodology on the one hand, and could stimulate students’ interest and make them armed with malaria knowledge and skills [4]. Meanwhile, to accelerate the process of malaria and achieve the goal of a “malaria-free” world, the WHO has designated 14 malaria-related collaborating centres globally, of which, five are training-oriented. These are: the Centre for Modelling, Monitoring and Training for Malaria Control and Elimination (Swiss Tropical and Public Health Institute, Switzerland), the Centre for Research and Training on Malaria (Ministry of Health, Myanmar), the Centre for Research and Training on Malaria Elimination (JIPD, China), the Centre for Case Management, Training and Research on Malaria (Mahidol University, Thailand), and the Centre for Training on Malaria Microscopy Diagnosis (Instituto de Diagnóstico y Referencia Epidemiológicos, Mexico). Only two of these WHO Collaborating Centres are focused on malaria elimination.

Despite a lot of efforts, moving toward the global goal of malaria elimination requires in-depth training in several critical issues, focusing on front-line field workers, entomologists, research scientists, and malaria-sensitive health system managers, policymakers and leaders [5].

China was once a country suffering from a serious malaria epidemic, with more than 30 million malaria cases annually before 1949 [6]. After decades of unremitting efforts using integrated malaria control strategy, China successfully achieved the goal of malaria elimination in 2021 [7]. Products, tools, experiences and malaria professionals were produced and gained [8], which can be tailored and used to contribute in accelerating global malaria elimination. For example, the anti-malarial drug artemisinin, discovered by Youyou Tu, has saved millions of lives globally. The “1-3-7” policy has been adapted to the local contexts of countries in Africa and Southeast Asia [7, 9]. China’s conduct of international training has also provided a venue for exchanging experiences and knowledge dissemination [10].

Health human resource development cooperation, an important part of China’s health international development assistance, refers to various forms of training and other personnel exchange projects for developing countries through multilateral and bilateral channels [10]. Malaria control has always been a key field and priority, especially after the launch of China-Africa Cooperation Forum in 2018 [11]. The Jiangsu Institute of Parasitic Diseases (JIPD) is one of the earliest specialized institutions for the prevention and control of parasitic diseases, including malaria and schistosomiasis in China. In 2002, JIPD was designated as an institution for foreign aid technical training by the Ministry of Commerce. Since then, the institute has held a number of technical training courses on malaria. To make full use of its role, the WHO designated the institute as a “WHO Collaborating Centre for Research and Training on Malaria Elimination” in October 2016. One major responsibility of the Institute as a collaborating centre is to conduct technical training and scientific research on malaria elimination strategies, measures and technologies. In this regard, JIPD needs to clearly understand the recipient countries' perception of the training and whether it matches their actual needs. This study has systematically reviewed the technical topics and methods used in the international training, using an online survey questionnaire to help improve future JIPD training programmes, to better contribute to the global efforts in eliminating malaria.

## Methods

### Research design and subjects

An assessment research framework was designed to evaluate the effectiveness of training courses, satisfaction of training participants, and to determine the directions for future international training. All individuals who participated in the training courses from 2017 to 2019 in JIPD were enrolled as research subjects and were invited to participate in the online survey.

### Questionnaire and data collection

A web-based questionnaire on the above assessment research framework was designed. Several experts and/or staff from JIPD and WHO provided inputs to the content of the questionnaire which was pre-tested to 15 randomly selected research individuals and revised accordingly. The following three aspects were included in the questionnaire: (i) basic information of respondents, (ii) evaluation of topics, methodology, trainers, facilitators, and impact of the course to the respondents, and (iii) suggestions and needs for improvement for future training. A score sheet evaluating the above topics as well as the overall impact of the course was tallied, with scores ranging from “0” to “5” with 5 being the highest degree of satisfaction or agreement.

Link to the web-based survey was distributed by e-mail to participants who attended the training courses in JIPD, Wuxi, China from 2017 to 2019. To obtain more feedback, the questionnaire was sent to each participant twice.

### Data analysis

Participants’ responses to the survey were downloaded, checked for completeness and consistency, entered into Microsoft Excel data sheets, and exported into Statistical Package for Social Science (SPSS) version 20 software for data analysis. Descriptive statistics were performed to measure relative frequencies and percentages. The arithmetic average was reported for continuous variables, as well as a bar chart to present the numerical distributions. High-charts were used to analyse the open-ended questions.

## Results

### JIPD’s journey on malaria international training

**Fig. 1:**
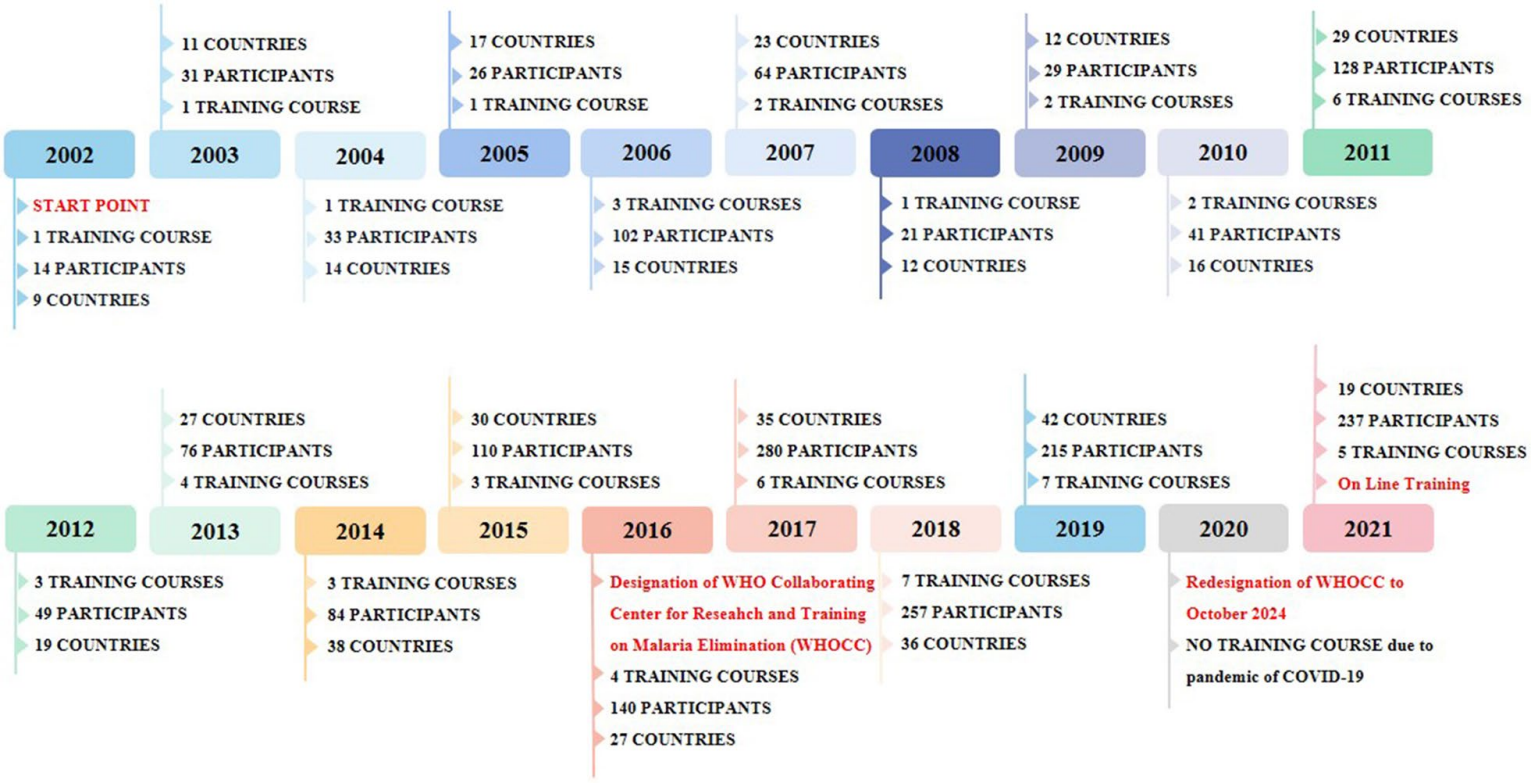
Footprint of international training by JIPD from 2002 to 2021

In 2002, JIPD was designated as an institution for foreign aid training of the Ministry of Commerce, China, and started the journey of malaria international training. JIPD has conducted 62 international training courses for 1935 participants from 85 countries worldwide since 2002 (shown in Fig. 1). Of 62 courses, 52 were about malaria, 8 were about infectious diseases (including malaria), and 2 were about schistosomiasis.

**Fig. 2:**
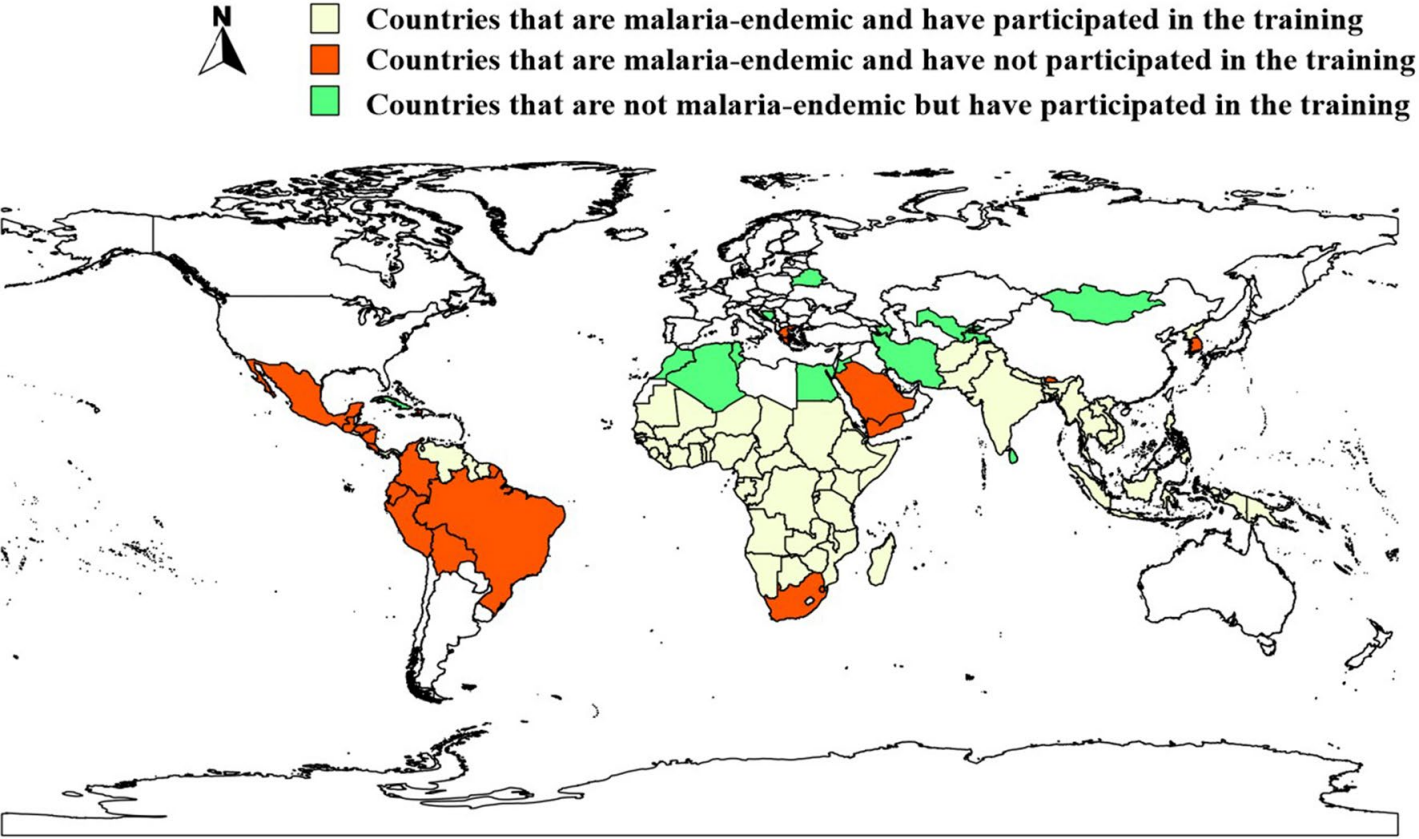
Distribution of countries that attended international trainings conducted by JIPD from 2002 to 2021

As indicated in Fig. 2, countries that attended international training courses conducted by JIPD from 2002 to 2021 included 42 (42/46) malaria endemic countries from Africa, 13 (13/17) from Asia, 5 (5/17) from the Americas and 2 (2/4) from Oceania.

## Assessment of participants’ satisfaction on malaria international trainings conducted by JIPD from 2017 to 2019

### General information

Of 752 participants who attended malaria training courses in 2017, 2018 and 2019 and had provided e-mail address, 170 volunteered to partake in the survey. General information of survey respondents is presented in Table 1. Respondents who participated in the English-speaking training accounted for 64.71%; of which 120 (70.59%) respondents were male; most were from African countries (70.00%), followed by Asia (22.35%); and with 108 (63.53%) respondents reporting that their country is currently in malaria control phase.

**Table 1:** Frequency distribution of the background characteristics of survey respondents (N = 170)

Characteristics	Frequency
	n (%)
Language	
English	110 (64.71)
French	60 (35.29)
Gender	
Male	120 (70.59)
Female	50 (29.74)
Region	
Africa	119 (70.00)
Asia	38 (22.35)
America	13 (7.65)
Year of training	
2019	61 (35.88)
2018	42 (24.71)
2017	67 (39.41)
Topic of training participated	
Malaria	106 (62.35)
Infectious diseases (including malaria)	64 (37.65)
Current status of malaria endemic nationally	
Elimination phase	62 (36.47)
Control phase	108 (63.53)

### Overall assessment score of training by survey respondents

Each respondent gave a score to evaluate individual satisfaction of the training course they attended on different aspects including course impact, topic, methodology, trainers and facilitators (Table 2). The average score on the overall usefulness of the training courses was 4.52 (5 maximum score). A majority of respondents reported that the knowledge and skills gained in the training courses were useful for their national malaria programme (4.28), the topics appropriate to their professional needs (4.52), and the knowledge and skills gained in the training useful to their career (4.52). On the methodology used, 153 respondents fully agreed (score of 4.83) that the methods in the training were enough to provide them with the necessary knowledge and skills needed to achieve the objectives. However, the average score on communication and presentation skills of the trainers and facilitators were relatively low (4.06), which means this needs improvements in future training.

**Table 2:** Assessment score of training courses by survey respondents

Variable	Score^a^						Average score^b^
	No answer	1	2	3	4	5	
Course impact							
Overall usefulness of the training courses	0 (0.00)^**c**^	5 (2.94)	1 (0.59)	4 (2.35)	51 (30.00)	109 (64.12)	4.52
Were the knowledge and skills you gained in the training courses useful for the national malaria program?	0 (0.00)	5 (2.94)	5 (2.94)	19 (11.18)	50 (29.41)	91 (53.53)	4.28
Were the topics covered in the training course relevant or appropriate to your role/job or professional needs?	0 (0.00)	3 (1.76)	4 (2.35)	7 (4.12)	43 (25.29)	113 (66.47)	4.52
Were the knowledge and skills you gained in the training useful to your career?	0 (0.00)	3 (1.76)	4 (2.35)	3 (1.76)	52 (30.59)	108 (63.53)	4.52
Course methodology							
Do you think the methods used in the training course enough to provide you with the necessary knowledge and skills needed to achieve the objectives of the training?	3 (1.76)	0 (0.00)	0 (0.00)	0 (0.00)	14 (8.24)	153 (90.00)	4.83
Trainers and facilitators							
Were the trainers and facilitators knowledgeable on the topics/subjects they covered or discussed?	3 (1.76)	3 (1.76)	3 (1.76)	6 (3.53)	59 (34.71)	96 (56.47)	4.37
Were the trainers and facilitators had good communication and presentation skills?	3 (1.76)	1 (0.59)	4 (2.35)	23 (13.53)	82 (48.24)	57 (33.53)	4.06

^a^The scores given by the respondents about their satisfaction of the training. The higher the score, the better the satisfaction.

^b^Average score: the sum of all scores marked by the respondents/the total number of respondents.

^c^N(%): the number of respondents by each score (the number of respondents by each score/the total number of respondents * 100%).

When asked the open-ended question “How did the training improve your national malaria programme”, the feedback of respondents mostly focused on the following aspects: clinical diagnosis and treatment, establishment of surveillance and response system, capacity building, prevention strategies. Below are some comments of respondents on the practical operation of translating knowledge into malaria control actions in their own countries after attending the training.

Respondent A from Vietnam: “*Application of 1-3-7 model in malaria foci investigation in Vietnam.*”

Respondent B from Senegal: *“The training has enabled me to better implement malaria control measures in our region, which is in the pre-elimination stage*.*”*

Respondent C from Sri Lanka: “*Although my country has eliminated malaria, imported cases are still coming to the country due to global malaria situation and the training is very useful to prevent them from re-spreading malaria in the country*.”

Respondent D from Ghana: “*The training helps our national malaria program by increasing the awareness of prevention and control of malaria by health educating the population on how to do so*.”

Respondent E from Afghanistan: “*I am working at the provincial level and the training was very useful for me, especially in-hospital control of malaria. Other Afghan participants are working at the national level. I’m sure that all of us used the knowledge of malaria control and elimination for developing the related policy of the ministry of public health Afghanistan*.”

### Evaluation of course topics and methodology by survey respondents

**Fig. 3:**
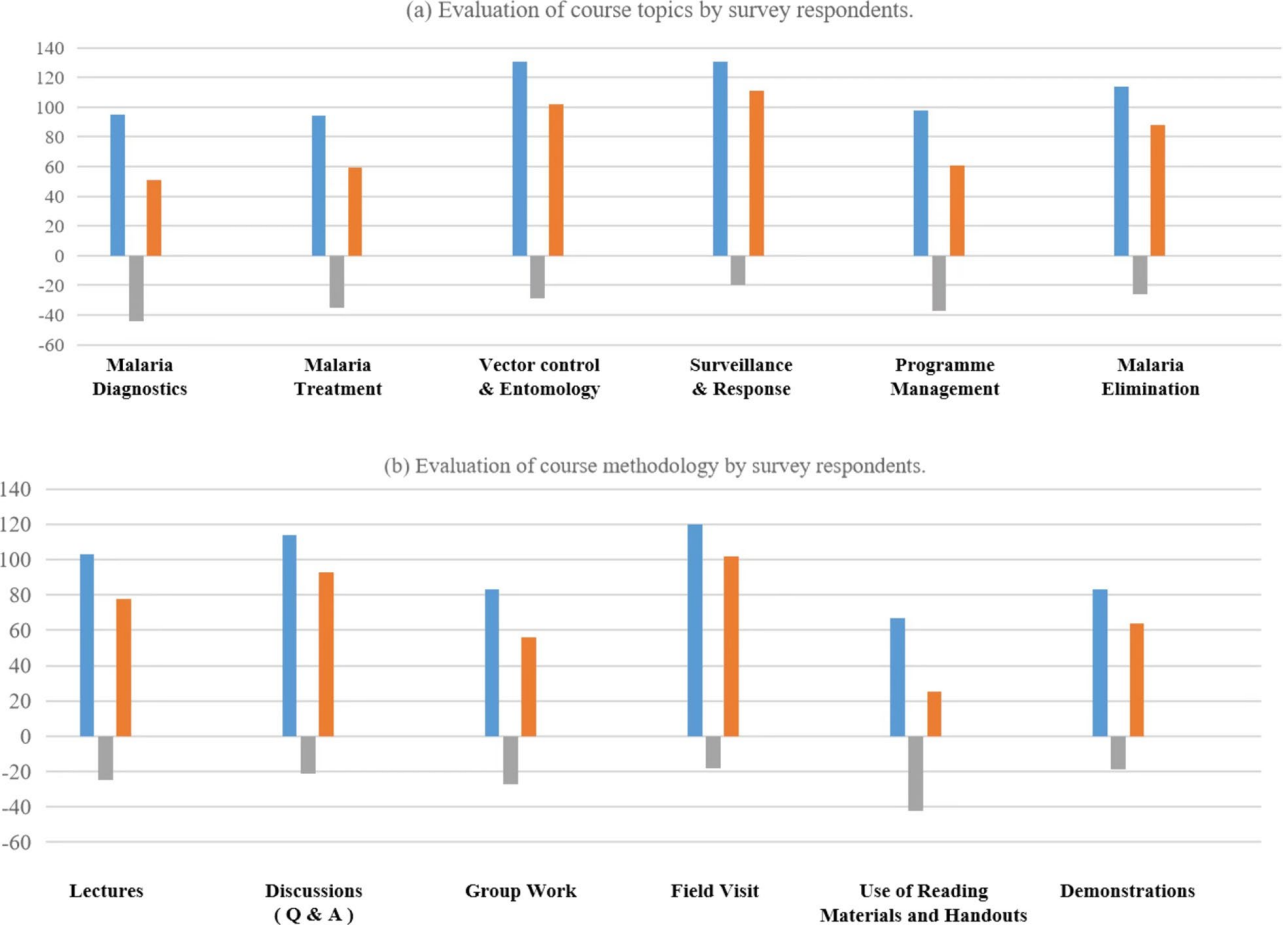
Evaluation of course topics (**a**) and course methodology (**b**) by survey respondents. Blue column: the number of respondents who think the topic / method was the most useful; Gray column: the number of respondents who think the topic/method was the least useful; Orange column: the number of respondents who think the topic/method was the most useful minus the number of respondents who think the topic/method was the least useful

Main technical areas covered in each malaria international training included theoretical and practical (i.e., field visits) applications. Technical areas include basic knowledge of malaria (diagnostics and treatment), national malaria elimination programme in China, strategies for vector control, principle and proposal for new anti-malarial drugs, progress on malaria vaccine and diagnosis, and management of Global Fund Project. For countries in pre-elimination phase, malaria surveillance and response was introduced as a main topic as well. This study investigated which of the areas covered in the training were most and least useful to the respondents. A majority of respondents agreed that they could benefit from nearly all the areas covered in the training, with only a small proportion of negative votes. Among all topics listed in the survey, the top three most useful areas were surveillance and response (111/170), vector control and entomology (102/170), and malaria elimination (88/170). However, the least useful area indicated was malaria diagnosis knowledge (51/170) (shown in Fig. 3a).

Field visits and demonstrations were also part of the training to better disseminate malaria related knowledge, stimulate participants' interest, and have a deeper understanding of China’s malaria control programme. These methods were most useful to the respondents (102/170), followed by discussions (93/170) and lectures (78/170). However, the least useful method according to the respondents was the use of reading materials and handouts (25/170).

### Suggestions on improving future malaria international training courses

**Fig. 4:**
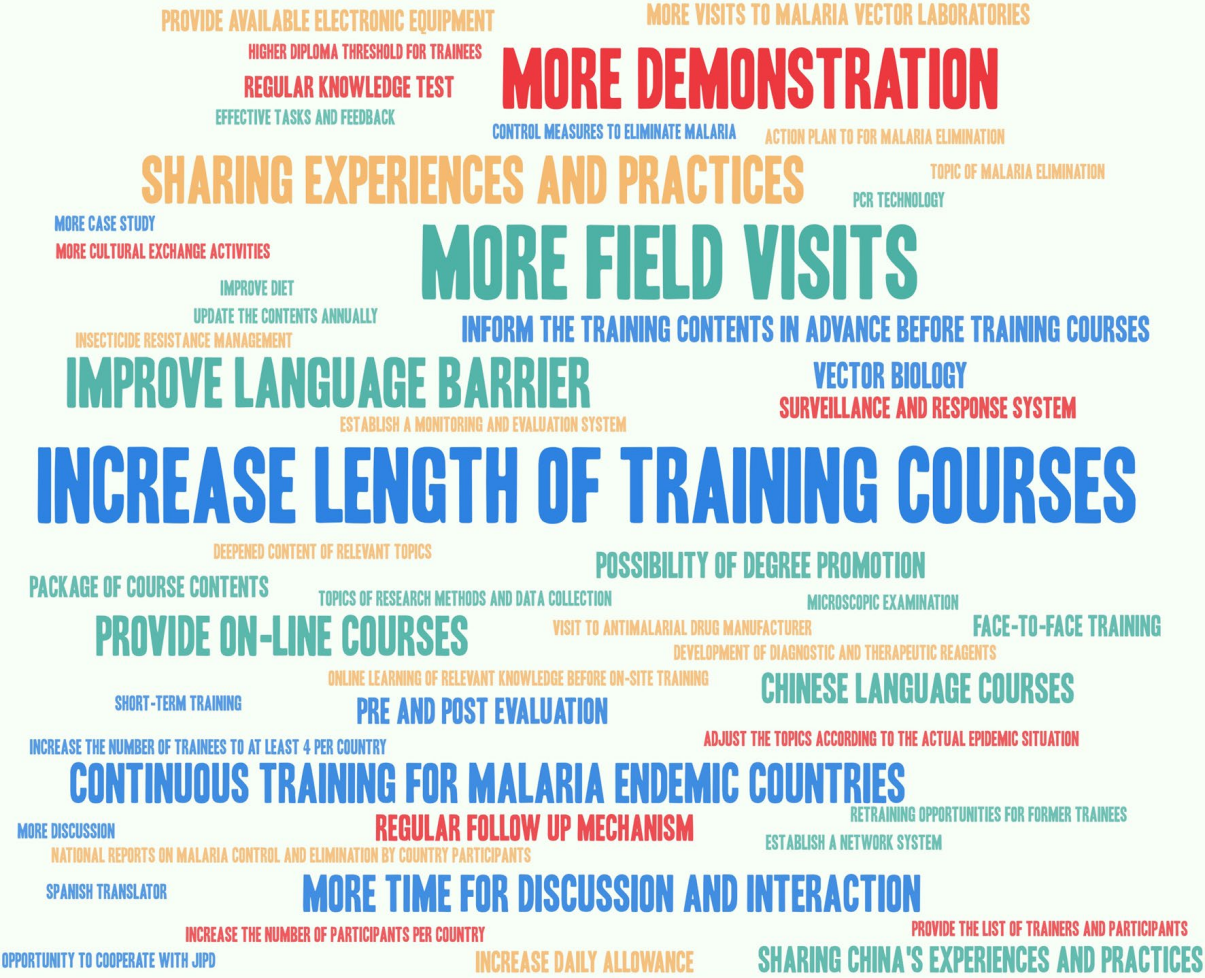
Highcharts of improvement suggestions for future international training by survey respondents

When asked “if you were to attend future training, what malaria topic/s or area/s would you like to be trained?” most respondents chose surveillance and response, elimination, and vector control. In the meantime, the respondents were asked to give suggestions on how to improve future trainings and results were summarized in Fig. 4.

In total, 36 respondents indicated that the training was good enough with no additional suggestions. Other suggestions were to prolong the length of training courses when possible to acquire more detailed information on malaria, as well as China; to organize 2 or 3 field visits to allow participants to have a better understanding and practical experience of malaria control and elimination in China; and more demonstration on microscopic examination, polymerase chain reaction (PCR), as well as mosquito identification and resistance determination. Improving language barrier was another suggestion by some respondents from French-speaking countries. Most trainers and facilitators from JIPD had fluent English communication skills, except for very few older malaria experts. However, for the trainings conducted in French, translators were employed but they did not have the necessary malaria background. Thus, some knowledge may have not been properly disseminated. Respondents also suggested that more experience and practical knowledge be shared from China’s malaria elimination programme, which may be applicable to other countries. Other aspects, such as on-line training, more spread of Chinese traditional culture, time for discussion and interaction, were suggested.

## Discussion

In the hope of strengthening the capacity-building of health professionals in malaria-endemic countries through international training and accelerate global malaria elimination, this study demonstrated the merits of conducting international training courses, understood the satisfaction and actual needs of trainees, and pointed out the direction for future improvements.

Currently, some countries and regions are still confronted with a high burden of malaria, mainly in Africa and Southeast Asia. The findings generally showed that international trainings on malaria conducted by JIPD since 2002 reached malaria-endemic countries in Africa, Asia and Oceania. Only a few countries in Latin America took part in the training, consistent with the overall aims of China's health assistance. With the continuous progress of global malaria elimination, many countries are facing a decrease in indigenous malaria cases and an increase in imported malaria cases [12]. Thus, training for malaria health workers in elimination settings is extremely important to maintain vigilance among all health practitioners to ensure early case detection and well-functioning malaria surveillance [13, 14]. It is gratifying that the training has also included countries that have already eliminated malaria or countries that have never had malaria epidemics historically, which is helpful to strengthen its professional and technical personnel for malaria control and even maintain the status of elimination.

In this survey, the scoring method found that most respondents gave a high score on its impact on their career, with field visits and demonstrations being the preferred methodology. Participants indicated that all aspects of the training were useful and believed that what they learned can improve their technical capacity on malaria.

Despite the satisfactory evaluation, there were suggestions and opinions put forward by respondents to improve future trainings. Firstly, increasing the duration of training. Usually, short-term on-job training, as the main mode of health human resource development cooperation, is mainly to provide training for officials above the junior level, so as to enhance their initial understanding of the problems associated with malaria control. Therefore, the duration of each training is generally 10–60 days, the forms of which include lectures, field visits. For deeper exchange and cooperation, degree education may be an alternative way [15]. Secondly, tailoring content specific to participants’ background and current country phase of malaria control or elimination. JIPD training participants come from different countries with different stages of malaria control or elimination. Thus, goals and strategies in different stages of malaria control vary greatly [16]. Also, participants did include clinicians, laboratory inspectors, entomologists, and even policy-markers from the Ministry of Health. Though various forms of training have been adopted including traditional lectures, it is still difficult to find a balance between training contents and methods tailored to each participant. Technical personnel may be more concerned about the diagnosis and treatment of malaria, while policy makers are about the construction of public health systems and the formulation of policies or strategies to eliminate malaria [2]. That is the reason why some respondents prefer more opportunities of field visits, whereas others prefer demonstration. In future trainings, it will and should be taken into consideration the actual needs of each training participant by administering a pre-training assessment. Thirdly, other country experiences particularly those certified as “malaria-free” by the WHO, should also be shared. China’s experiences in malaria elimination can be tailored and used in countries with high malaria burden [17]. However, sharing other countries’ experience in fighting malaria can also be an important component in future trainings.

Also, approximately 79% of the participants deemed that it would be better to establish an informal communication network between trainers and participants. Ideas for the activities of the network could include but not limited to the following aspects: (i) sharing the progress and experience of countries in eliminating malaria; (ii) disseminating the latest research progress and the technical guidelines of the WHO; (iii) conducting international training or seminars regularly or irregularly; and (iv) brainstorming, communication platform, case sharing. This proposal of establishing a network platform will be taken into consideration to facilitate sharing of different country experiences, identifying the needs of recipient countries, and disseminating WHO guidelines.

Since 2020, the rapid emergence and spread of corona-virus disease 2019 (COVID-19) worldwide has caused substantial global disruptions that are impacting health programmes including malaria [18]. Resources have been re-allocated for the prevention and control of COVID-19, leading to re-emergence of malaria burden [19]. Unfortunately, COVID-19 pandemic has also affected health development assistance, including conduct of face-to-face international training courses. In 2020, no malaria-related training courses were held in JIPD. To maintain the continuity of training, five online training courses were developed for 237 participants from 19 countries.

The pace of capacity building through training should not be stopped regardless of the malaria epidemic in the country. Some countries understand the difficulties and challenges faced by them through training and evaluation in malaria health workers to find appropriate solutions, including Nigeria [20], Nepal [21], India [22], China [14]. After the launch of China-Africa Cooperation Forum, capacity building on malaria has been a priority and is high on the agenda. China will and should contribute its wisdom to the global efforts to eradicate malaria, and jointly build a community of shared future for mankind.

## Conclusions

Offering health human resource training opportunities has been an important part of China’s health development assistance for developing countries. JIPD, as a professional institute for malaria control, has conducted a great quantity of training in the past 20 years, and achieved remarkable achievements and has contributed to malaria elimination efforts. In the future, international training will be conducted considering the suggestions for improvement to better contribute to the global process of malaria elimination.

## Data Availability

All data generated or analysed during the study are included in this article, and please contact author for data requests.

## Abbreviations

WHO: World Health Organization
JIPD: Jiangsu Institute of Parasitic Diseases, China
WHOCC: WHO collaborating centre
